# Editorial: Community series in mental illness, culture, and society: dealing with the COVID-19 pandemic: volume V

**DOI:** 10.3389/fpsyt.2023.1205905

**Published:** 2023-05-10

**Authors:** Samer El Hayek, Renato de Filippis, Mohammadreza Shalbafan

**Affiliations:** ^1^Medical Department, Erada Center for Treatment and Rehab in Dubai, Dubai, United Arab Emirates; ^2^Psychiatry Unit, Department of Health Sciences, University Magna Graecia of Catanzaro, Catanzaro, Italy; ^3^Mental Health Research Center, Psychosocial Health Research Institute, Department of Psychiatry, School of Medicine, Iran University of Medical Sciences, Tehran, Iran

**Keywords:** lockdown, mental health, mental disorders, psychiatry, psychological impact, SARS-CoV-2, social distancing, social isolation

The COVID-19 pandemic has caused, not only serious socioeconomic consequences, but also physical, psychological, and mental health crises (1). Since the start of the pandemic, high rates of psychological distress, depression, anxiety, and post-traumatic stress were reported (2), and numerous efforts have been made to reduce the treatment gap by addressing the mental health requirements of underprivileged communities and nations (2, 3). Risk factors include female sex, student status, unemployment, and physical and psychiatric comorbidities (4). This impact of the pandemic is also moderated by other variables, such as ethnicity (5), culture (6), and being part of vulnerable population groups (7).

Following the previous four volumes of our Community Series Research Topic entitled “*Mental illness, culture, and society: dealing with the COVID-19 pandemic*” (8–11), this fifth volume features nine new papers that investigated the relationship between mental health and the COVID-19 pandemic in specific populations and communities.

Two studies looked at the impact of the pandemic on the mental health of young individuals. Liu et al. examined depressive symptoms in 2,554 postgraduate students residing in eastern China. Collected data included the Patient Health Questionnaire, the interpersonal sensitivity subscale of Symptom Checklist-90, the Psychological Capital Questionnaire, and the Pittsburgh Sleep Quality Index. The prevalence of mild, moderate, and severe depressive symptoms was 30.97, 6.58, and 1.45%, respectively. Psychological capital and sleep quality independently mediated the relationship between interpersonal sensitivity and depressive symptoms (indirect effect = 0.136 and 0.100, respectively, *p* < 0.001) and together co-played a chain-mediating role (indirect effect = 0.066, *p* < 0.001). The authors concluded that positive psychological interventions and sleep guidance may be beneficial in alleviating depressive symptoms in this population. In another cross-sectional study, Wong, Nik Farid et al. looked at how 561 young individuals aged 18–24 years from low-income communities were responding to the pandemic. The questionnaire included the Parental Environment Questionnaire (PEQ), the Brief Resilient Coping Scale (BRCS), and the Depression, Anxiety, and Stress Scale-short form (DASS-21). The prevalence of depression, anxiety, and stress were 12.5%, 15.2%, and 6.4%, respectively. Parent-child conflict was the strongest significant predictor for higher levels of depression (OR = 10.90, 95% CI 4.31–27.57), anxiety (OR = 11.92, 95% CI 5.05–28.14), and stress (OR = 4.79, 95% CI 1.41–16.33) symptoms. Females and those from low-income households had more severe symptoms of depression and anxiety. Furthermore, those employed had greater severity of anxiety symptoms compared to those unemployed, while a lower level of physical exercise was associated with higher depressive symptoms.

Likewise, Wong, Alias et al. recruited 553 parents of children aged 13–24 years from low-income community settings and assessed the parent-child relationships using the PEQ and DASS-21. Married parents reported a higher level of parent-child conflict than single parents (OR = 3.18, 95% CI 1.30–7.75). More parent-child conflict was noted in participants aged 60–72 years old who were unemployed, retired, housewives, or from lower-income groups. Alternatively, physical activity and enough sleep were associated with a lower level of conflict. The authors suggested that this low risk of parent-child conflict and psychological sequelae for parents could be due to numerous support measures implemented by the government.

Three studies looked at different patient populations during the pandemic. Thirunavukkarasu et al. evaluated oral health-related quality of life (OHRQOL) and its association with mental health among 677 patients with type 2 diabetes mellitus (T2DM) in Saudi Arabia. For their assessment, the authors used the Arabic version of the Oral Health Impact Profile-14 questionnaire and DASS-21. Half of the participants (52.7%) had poor OHRQOL. This was significantly higher in patients with a longer duration of T2DM (aOR = 3.31, 95% CI 1.96–4.17) and those who did not periodically monitor their oral health (aOR = 2.85, 95% CI 1.76–3.89). Total OHRQOL scores had a significant association with depression (aOR = 2.32, 95% CI 1.34–3.71, *p* = 0.001), anxiety (aOR = 1.81, 95% CI 1.22–2.79, *p* = 0.003), and stress (aOR = 1.43, 95% CI 1.14–2.19, *p* = 0.026). Findings highlight the importance of health education programs for patients with T2DM to ensure improved both oral and mental health outcomes. Zhang et al. compared the electronic medical records of patients visiting the largest psychiatric emergency department in China in 2020, compared to before the pandemic. Compared to 2018 and 2019, the proportions of visits related to anxiety and stress disorders in 2020 significantly increased (from 83 in 2018 to 239 in 2020; 188.0% increase) and patients were significantly younger (*p* < 0.001). Findings highlighted the need for well-equipped crisis prevention services during the pandemic. Wu et al. examined perceived COVID-19 stigma using the Short Version of COVID-19 Stigma Scale (CSS-S) in 1,297 patients who recovered from COVID-19 in China. The authors identified three profiles of perceived COVID-19 stigma: low (12.8%), moderate (51.1%), and severe (36.1%). Older age, living with other people, anxiety, female gender, and sleep disorder were positively associated with moderate and severe perceived COVID-19 stigma. Alternatively, higher education, social support, and peace of mind were negatively associated with severe perceived stigma. The authors identified the value of ≥20 as an optimal cut-off for the CSS-S.

Two studies looked at mental health during pregnancy or after delivery. Guo et al. explored the impact of the pandemic on two cohorts of pregnant women at their first antenatal care in China (pre-COVID-19 group, *n* = 5,728 and COVID-19 group, *n* = 739). The Patient Health Questionnaires-9 and −15 and Generalized Anxiety Disorder-7 scale were used to assess symptomatology. There were significant differences in the demographic characteristics between the two groups (*p* < 0.05) and newly registered participants for antenatal care dropped by about 50% during the pandemic. After matching demographics, the prevalence of depression, generalized anxiety disorder, and somatoform disorder was found to be significantly higher during the COVID-19 pandemic compared to before (2.3%, 9.6%, 20.8% vs. 0.3%, 3.9%, and 10%, respectively). The authors concluded that the pandemic not only increased mental health problems among pregnant women but also decrease antenatal care clinic attendance. Zou and Chen examined the emotional status of 36 new mothers with infants aged 0–1 years old in China. In this qualitative study, the authors found participants to be chronically depressed, feeling anxious, and upset. Negative emotions were caused either by COVID-19 or by the strict epidemic control policy implemented in China. The new mothers were also anxious about their offspring's physical health, feeding options, and childcare. Lastly, positive emotions were related to strong parent-child bonds, a better understanding of childcare, and a good ability to perceive risks.

Lastly, Kin Ng et al. looked to validate the traditional Chinese versions of the 36-item and 18-item COVID Stress Scales (CSS-36 and CSS-18, respectively) in Hong Kong. The study included 521 undergraduate students (61% female, aged 18–26 years). Findings offered evidence for the psychometric properties of the scales in the Hong Kong context. The results of confirmatory factor analyses supported a six-factor structure for both the CSS-36 and the CSS-18. In addition, both scales exhibited good internal consistency, reliability, and concurrent validity with fear of COVID-19 and negative emotional states.

In conclusion, the articles collected in the fifth volume of our Research Topic further highlight the impact of COVID-19 on different population groups. Mental health professionals must collaborate to offer prompt and customized treatment services to impacted individuals, especially those who are considered to be at a higher risk.

## Author contributions

All authors listed have made a substantial, direct, and intellectual contribution to the work and approved it for publication.
